# Structure–Property Relevance of Two Pairs of Isomeric Steviol Rebaudiosides and the Underlying Mechanism

**DOI:** 10.3390/foods14111917

**Published:** 2025-05-28

**Authors:** Zhuoyu Zhou, Wanjie Wang, Qinbing Guo, Haijun Wang, Yongmei Xia

**Affiliations:** 1 Key Laboratory of Synthetic and Biological Colloids, School of Chemical and Materials Engineering, Jiangnan University, Wuxi 214122, China; 7200610016@stu.jiangnan.edu.cn (Z.Z.); wanjie0504@163.com (W.W.); wanghj@jiangnan.edu.cn (H.W.); 2 State Key Laboratory of Food Nutrition and Safety, Tianjin University of Science and Technology, Tianjin 300457, China; guoqingbin008322@tust.edu.cn

**Keywords:** rebaudioside D, rebaudioside A, transglycosylation, alternansucrase, edulcorant, physiochemical properties

## Abstract

Although enormous efforts have been made to prepare tasty and soluble steviol glycosides (SGs), the structure–property relationship of SGs still remains unclear, neither in experiment fact nor in the mechanism, such as the influence of linkage type and position of substituted glucosyl on physiochemical properties and sensory features of SGs. The favorable SGs, rebaudioside D (RD) and rebaudioside A (RA), possess good edulcorant quality, poor solubility, and other significantly different physical properties. This research chose two pairs of isomeric SGs, RA and its isomer rebaudioside E (RE) and RD and its isomer RA1G (a synthetic SG, *α*-1,6-mono-glucosylated RA), to conduct a comparative study, aiming to reveal the structure–property relevance on their solubility, sweetness, stability, and crystal structure. The RA1G presents an aqueous solubility 13 times that of RA and 137 times that of RD and exhibits better edulcorant quality than that of RA, similar to RD. The results indicate that the glucosyl linkage type and position have a stronger impact on the properties of the SGs than the number of glucosyl moieties. The underlying mechanism of their structure–property relevance was elucidated by analyzing the interaction energies between the SGs with solvent and human receptor proteins, respectively.

## 1. Introduction

For decades, many efforts have been made to obtain tasty and functional steviol glycosides (SGs) for sugar substitution [1,2,3,4]. In addition to sweetness and solubility, other physiochemical properties such as solution interfacial properties, stability, and crystalline properties of SGs are also critical for the storage and application of SGs. However, except for the knowledge that more glycoside moieties on an SG molecule cause less sweetness and bitterness [5,6], the structure–property relationship of SGs still remains unclear [7], especially regarding the influence of linkage type and position of the glycosyl moieties on the physiochemical properties and sensory feature of SGs, which is obviously crucial for both the application and synthesis design for new SGs.

**Scheme 1:**
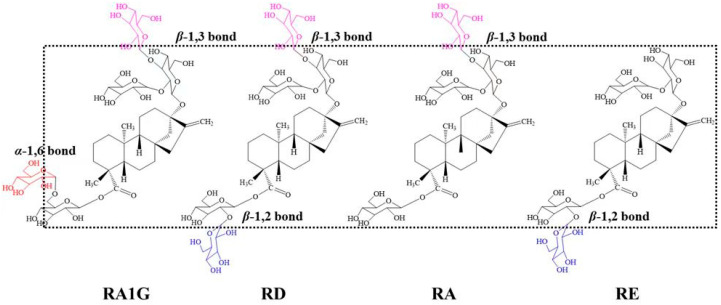
Structure of the isomeric SGs: RA1G (synthesized in this work) and RD, RA, and RE.

Rebaudioside D (RD), a well-known steviol glycoside (SG), has been considered one of the best natural sweeteners. However, its low solubility is a barrier to its application in foods and drinks [8,9,10]. Meanwhile, rebaudioside A (RA) is the most popular SG used in food and drinks due to its high sweetness and better solubility compared to that of RD, but it has often been criticized for its bitter aftertaste [11], which is a reason for using RD as an alternative to RA. Structurally, RD is differentiated from RA by an additional glucose moiety at the C-19 position through a *β*-1,2-glycosidic linkage (Scheme 1). This modification provides less bitterness than RA does; however, it is not expected that the extra hydrophilic glucose moiety actually causes RD to possess a lower solubility than RA does.

The glucosylation of RA [12,13,14] or St (stevioside) [15,16] with different linkages can provide a better taste profile and higher solubility compared to the starting SG. In general, the glycosylated products contain isomeric RD (various mono-glucosylated RA, i.e., RA1G) or isomeric RA (St1G) and many multi-glucosylated products. For example, *α*-glucosyl-rebaudioside A (RAnG) obtained with Gtf180-ΔN glucansucrase [12,13,17], or *α*-glucosyl-stevioside (StnG) synthesized with an alternansucrase [15,16].

The natural isomeric RA, like rebaudioside E (RE), exhibits similar polarity to RA (the log *p* of RA and RE is −1.45 and −1.43, respectively, calculated with ALOGPS 2.1), implying a similarity in their aqueous solubility (still lack of solubility data). However, it is interesting that RA possesses a sweetness twice that of RE [18], which conflicts with the commonly known role of the sweetness-structure relationship of SGs—weaker sweetness coming from more glucosyl moieties attached to the C13 position of the steviol backbone, implying that C13 substitution to the steviol backbone might result in weaker sweetness than C19 substitution, which could be explained either by a comparative study on the available isomers or by computational simulation to verify the mechanism or in case there are no available isomers.

Therefore, it would be interesting to know how and why one more glucosyl moiety in SG molecules would influence the solubility, sweetness, and other physicochemical properties.

In this study, we synthesized an isomer of RD by introducing an *α*-glycosidic linkage to RA under the catalysis of an alternansucrase, namely RA1G, making a sample of an isomer of RD. In addition to the isomer pair of RD and RA1G, another pair of isomers (RA and RE) was taken into the comparative study on their sweetness, solubility, stability, and crystal structure. This analysis aims to partially but closely elucidate the impact of glycosyl moieties and glycosidic linkages on the physicochemical properties of SG.

## 2. Materials and Methods

### 2.1. Materials

Alternansucrase derived from *L. citreum* CICC23234 was a gift from Professor Jin Wu at Jiangnan University, Wuxi, China. Rebaudioside A (HPLC purity of 97%), rebaudioside D (HPLC purity of 97.8%), and rebaudioside E (HPLC purity of 95%) were from Zhucheng Haotian Pharmaceutical Co., Ltd., Zhucheng, China. Sucrose and other reagents were of analytical grade, supplied by Sinopharm Chemical Reagent Co., Ltd., Shanghai, China, and used as received unless stated specifically.

### 2.2. Preparation and Characterization of α-1,6-Glycosylated Rebaudioside A

To obtain isomeric RD (RA1G) for the comparative study, an alternansucrase derived from *L. citreum* CICC23234 was applied to catalyze the glucosylation of RA with sucrose. Reaction conditions were optimized to harvest RA1G for characterization and property study. The raw product was separated and purified with column chromatography [19]. The structure characterization of the RA1G was carried out with HPLC, methylation analysis, MS, and 1D and 2D NMR analysis [19,20].

HPLC analysis: The resulting products were dissolved in methanol and analyzed using an HPLC system (Waters 2695, Milford, MA, USA). The data were recorded from a Lichospher NH_2_ column (4.6 mm × 250 mm, 5 μm, Hanbon Sci. & Tech., Shanghai, China), with acetonitrile aqueous solution for gradient elution: 80% (*v*/*v*) of acetonitrile for 0–2 min, 80% to 50% of acetonitrile for the next 3–18 min, and then back to 80% of acetonitrile for another 5 min at 1 mL/min.

The RA conversion and the yield of glucosylated rebaudioside A (RAnG, denoted for n mol of glucose substituted on 1 mol of RA) were calculated as follows, respectively,

(1) $$RA\;conversion\left(\%\right)=\frac{C_{0}-C_{t}}{C_{0}}\times 100\%$$

(2) $$RAnG\;yield\left(\%\right)=\frac{C_{RAnG}/{MW}_{RAnG}}{C_{0}/{MW}_{RA}}\times 100\%$$

where *C_t_* (g/L) and *C_RAnG_* are the concentration of RA and RAnG in the reaction mixture at time *t*, *C*_0_ (g/L) is the initial RA concentration, respectively. MW_RA_ and MW_RAnG_ are the molecular weights of RA and RAnG, respectively. The RAnG yield was determined based on its HPLC area calibrated with that of RA.

Mass Analysis: The molecular weight of each product was determined using LC-MS using a Waters Acquity UPLC coupling with a Waters Maldi Synapt Q-TOF MS. Collision energy, 6 eV. The gradient eluent (0.8 mL/min) was set up as the following: 80% (*v*/*v*) of acetonitrile for the initial 10 min, followed by a transition from 60% to 40% of acetonitrile over the next 10 min, maintaining 40% acetonitrile for the subsequent 7 min, and finally reaching 100% acetonitrile for another 27 min.

NMR Analysis: The 1D (^1^H and ^13^C) NMR spectra were recorded as per the literature [20,21]. Homonuclear ^1^H/^1^H (COSY, TOCSY) and heteronuclear ^1^H/^13^C correlation (HSQC, HMBC) were performed for linkage analysis of glycosylated SG [19]. Briefly, the 1D (^1^H and ^13^C) NMR spectra were recorded on a Bruker Avance III HD 600 spectrometer (Bruker, Faellanden, Switzerland) equipped with a 5 mm TCI cryoprobe, using deuterated methanol (CD_3_OD) as the solvent. The spectra were acquired at 600 MHz for ^1^H and 151 MHz for ^13^C at a probe temperature of 25 °C. Chemical shifts were referenced to the internal CD_3_OD signals at 3.30 ppm for ^1^H and 49.0 ppm for ^13^C spectra, respectively.

### 2.3. Determination of Solubility and Calculation of Solvation Free Energy of the SGs

The solubility in water of SGs was tested according to the literature [22] with slight modification. First, 0.5–1 g of steviol glycosides (SGs) in 10 mL of deionized water was stirred at 25 °C for 24 h. The resultant suspension was centrifuged, and the supernate was filtered through a syringe filter (0.22 μm) for HPLC analysis to determine the sample concentration at over-saturation, which corresponds to the solubility.

The calculation of the solvation free energy of the four SGs was executed with Gromacs 2018.8 [23]. The topology files of SGs were generated with SOBTOP 1.0. The change in free energy, ΔG, is a function of a coupling parameter λ. In the simulations, 20 linearly varying λ values were set to obtain the ∂H/∂λ curve. For each λ value, a sequence of steepest descents minimization, NVT equilibration, NPT equilibration, and data collection under an NPT ensemble was performed. The sum of ΔG across all λ conditions yields the solvation free energy.

### 2.4. Crystal Properties Measurement

Sample preparation: A total of 50 mg of purified SG sample was dissolved in 0.5 mL of 80% methanol and refluxed for 10 min under magnetic stirring. After immediate filtration, the filtrate was left to crystallize at 4 °C for 24 h. The resultant solid was then dried under vacuum at 4 °C.

XRD (X-ray powder diffraction) analysis: XRD analysis was conducted using a Bruker D8 Advance Davinci diffractometer system (Bruker AXS, Madison, WI, USA). The diffraction pattern was collected at room temperature over an angular range of 5° to 60°.

DSC (differential scanning calorimetry) analysis: DSC analysis was performed using a DSC 204 F1 instrument (NETZSCH Scientific Instruments Trading Ltd., Shanghai, China) equipped with a liquid nitrogen cooling system. The melting curves of SG crystals were measured within a temperature range of 150–300 °C at a scanning rate of 10 °C/min.

TGA (thermal gravimetric analysis) assay: TGA was carried out using a TGA/DSC1/1100SF instrument (METTLER TOLEDO, Zurich, Switzerland) at 20 °C/min.

Polarizing microscope characterization: Observation was carried out using a thermal platform microscope (Axio Imager A2POL, Carl Zeiss, Deutschland, Germany) at 500× *g* magnification at 25 °C and a polarization angle of 70°.

### 2.5. Solution Interfacial Properties Determination and Calculation

Solution interfacial properties of the SGs were determined and calculated according to the literature [6]. The surface tension/concentration curve of the SG aqueous solution was measured at 25 °C using the Du Noüy ring method with a K100 automatic surface tensiometer from KRÜSS, Germany.

### 2.6. Sensory Evaluation

A sensory evaluation panel was comprised of people aged 22 to 30 (6 females and 4 males). The panelists were graduate students of Food Science and Technology at Jiangnan University who consented to participate in the study [7]. The whole study was approved by the Jiangnan University Medical Ethics Committee (Reference Number: JNU202406RB024).

The sample for sensory evaluation was a 0.02% SG aqueous solution. Each evaluator took a sip of the solution (about 1 mL), held it on the tongue for 7 s, then spat it out and rinsed the mouth 3 to 5 times with 250 mL of pure water. Subsequently, each evaluator rated the SG solution for sweetness, bitterness, astringency, cooling sensation, metallic taste, after-sweetness, after-bitterness, after-astringency, and licorice aftertaste. Scores from the ten evaluators were averaged to derive the sensory ratings for each SG sample.

### 2.7. Estimation of Interaction Energy Between Each SG with Receptor Proteins Toward Edulcorant Property

To understand the mechanism of the structure/edulcorant property, the interaction energies between SGs with receptor proteins (sweet taste receptors hT1R2 and hT1R3, and bitter taste receptor hT2R4) toward edulcorant property were estimated with a similar approach as described in our previous work [21] and the literature [7,24].

### 2.8. Determination of Stability of the SGs in Digestion Fluids

Samples were dissolved in simulated digestion fluids and subsequently incubated in a water bath at 37 °C for 8 h. The starting concentration of each SG was 0.2 mg/mL. Sample concentration after digestion was analyzed with HPLC.

A stomach simulation solution was prepared using 0.04 mol/L hydrochloric acid (pH 1.5). Intestinal simulation solution: prepare a 5 mmol/L solution of potassium dihydrogen phosphate and adjust the pH to 6.8 using a 0.1 mol/L sodium hydroxide solution, then dilute the solution with distilled water to double the volume.

### 2.9. Statistical Analysis

All experiments were conducted with three replicates. Data were expressed as mean ± SD values.

## 3. Results

### 3.1. Preparation and Characterization of the Isomer of RD (RA1G)

An RD isomer (RA1G) was prepared to construct the isomer system for the structure–function relevance study. As mentioned above, RA1G can be in the products (*α*-1,6-glucosylated RA) of transglycosylation catalyzed by wild-type Gtf180-ΔN glucansucrase [12] or as a tri-glucosyl stevioside yielded from stevioside and sucrose in aid of an alternansucrase [16] or glucansucrase [17].

**Figure 1:**
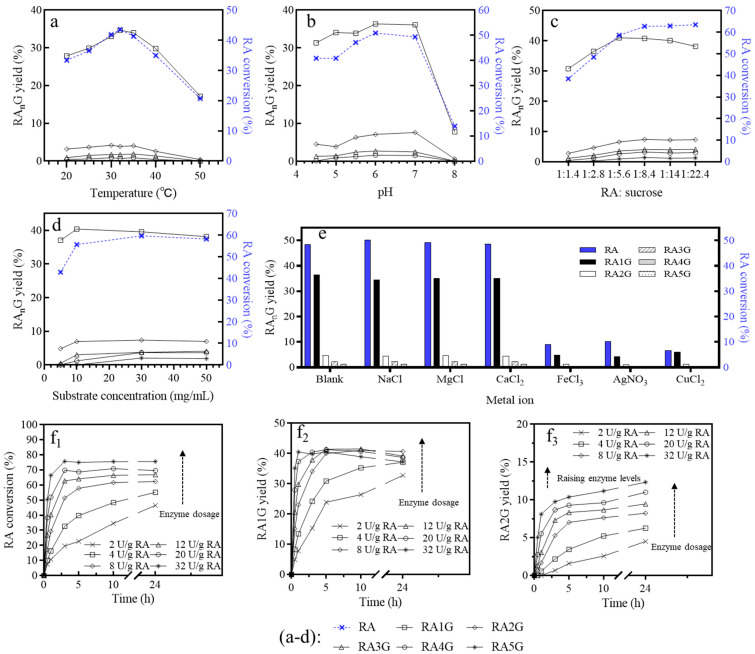
Transglycosylation of rebaudioside A catalyzed by the alternansucrase from *L. citreum* CICC23234. (**a**) Temperature effect: RA/sucrose (mol/mol) = 1:2.8, 10 mg sucrose/mL, 8 U/g sucrose, 3 h. (**b**) pH effect: 20 mM citric acid sodium citrate buffer. RA/sucrose (mol/mol) = 1:2.8, 10 mg RA/mL, 32 °C, 8 U/g RA, 5 h. (**c**) Effect of substrate ratio: 10 mg RA/mL, 8 U/g RA, 32 °C 5 h. (**d**) Effect of substrate concentration: RA/sucrose (mol/mol) = 1:5.6, 10 mg RA/mL, 8 U/g sucrose, 32 °C, 5 h. (**e**) Effect of metal ion: 5 mmol/L metal ion, RA/sucrose (mol/mol) = 1:2.8, 10 mg RA/mL, 8 U/g RA, 32 °C,5 h. (**f**) Effect of enzyme dosage on RA conversion (**f1**), RA1G yield (**f2**), and RA2G yield (**f3**); RA/sucrose (mol/mol) = 1:5.6, 10 mg RA/mL, 32 °C.

However, the reported cases were not ideal for harvest-purified RA1G because of the availability of enzymes and the insufficient yield or structure of RA1G for this research. The structure similarity of the isomer is favored herein, like *α*-1,6-glucosylated RA. In our experiment, an alternansucrase was employed to convert RA to RA1G (Figure 1). Subsequently, to maximize the yield of isomeric RD, the reaction conditions were optimized (Figure 1a–f). The alternansucrase was also chosen because it is an *α*-transglucosylase known for efficiently synthesizing alternan with alternating *α*-1,3 and *α*-1,6 linkages [25,26], such as uncharacterized tri-glucosyl-stevioside [16], and an *α*-1,6-glucosyl rebaudioside C [27].

The alternansucrase exhibited higher activity at lower temperatures, resulting in the highest RA conversion rate (43.56%) and RA1G yield (37.67%) at 32 °C (Figure 1a), with optimal pH range between pH 5.5 and 7 (Figure 1b).

The ratio of sucrose-to-RA played a significant role in shaping the distribution of RA substitution. The conversion rate reached the highest when the molar ratio of RA-to-sucrose was 1:8.4 (mass ratio of 1:2.97), indicating that excess glucosyl donors actually inhibited glucosylation (Figure 1c). For *α*-glucosylation of stevioside (St) with sucrose and the Gtf180-ΔN-Q1140E mutant enzyme [17], the highest conversion rate of St was achieved at the St/sucrose ratio (mol/mol) of 1:16.9. Herein, the maximum yield of RA1G was obtained at the molar ratio of RA-to-sucrose of 1:5.6. Higher substrate concentrations resulted in a significant increase in the production of tri- and tetra-substituted products (Figure 1d). In addition, Na^+^, Mg^2+^, and Ca^2+^ exhibited a marginal enhancement in RA conversion, while Fe^3+^, Ag^+^, and Cu^2+^ exerted pronounced inhibitory effects on the transglycosylation activity of the alternansucrase (Figure 1e). However, more enzymes provided a higher yield of multi-substituted products but low selectivity of RA1G, requiring precise control of enzyme dosage (Figure 1(f1,f3)).

In short, the maximum yield of RA1G was over 40% in 1 h with an enzyme dosage of 32 U/g RA (Figure 1(f2)), or 8 U/g RA in 5 h, where the RA conversion was about 75.8%. In another work, a stevioside conversion of 43.7% was obtained using 10 mg/mL of sucrose and an alternansucrase at 100 U/g St [16] in 24 h, which yielded a mixture of various isomeric mono-/di-/tri-glucosylated St.

Molecular structure identification of the RA1G was carried out with methylation analysis (Figure S1, Table S1) and NMR analysis (Figures S2–S4), including ^1^H (Figure S2), ^13^C NMR (Figure S3), as well as 2D NMR profiles (Figure S4a–e). All characterization profiles indicate that the alternansucrase yields the same RA1G (Scheme 1) as the Gtf180-ΔN-Q1140E enzyme [12].

### 3.2. Polarity, Solubility, Solution Interfacial Activity of the Isomeric Steviol Glycosides

**Figure 2:**
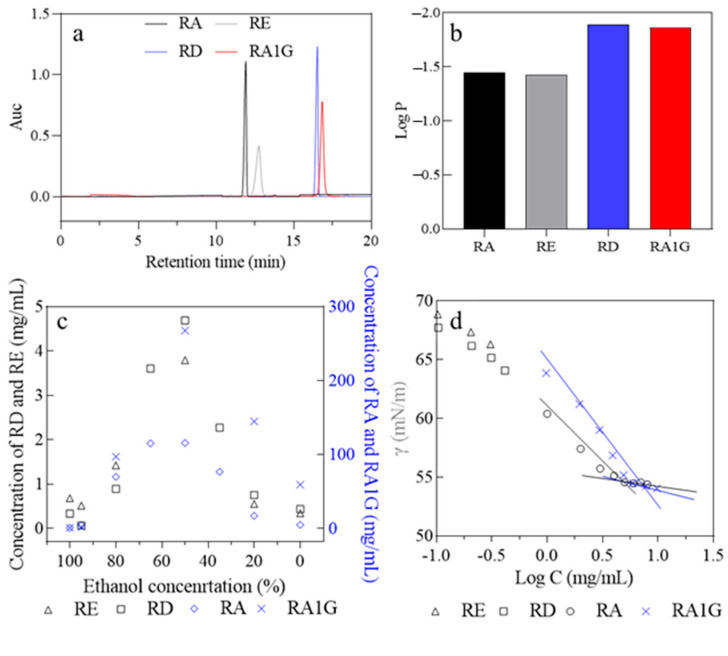
The properties of the SGs solution at 25 °C. (**a**) HPLC profile. (**b**) Log *p*. (**c**) Solubility in ethanol/water solution. (**d**) γ-Log C.

In normal-phase chromatography, the substances with shorter retention times have lower polarity if their molecular weights are the same. The polarity order of the SGs is based on the retention time followed by RA < RE, RD < RA1G (Figure 2a). However, the calculated Log *p*-values of RA, RE, RD, and RA1G are −1.45, −1.43, −1.89, and −1.86, respectively (calculated with ALOGPS 2.1), which are ranked against the order of retention time. This inconsistency in the datasets may be due to the insignificance (*p* > 0.05) of Log *p*-values inside the isomeric steviol glycosides (Figure 2b).

The difference in polarity between the isomeric SGs suggests that the glucosyl group at the C19 position of the aglycone (RE, RA1G) yields higher polarity than the glucosyl group at the C13 position (RA, RD). Generally, the polarity of a substance is also used to predict its solubility.

It is interesting that RD and RA1G are the molecules that possess a hydrophilic glucosyl group at the C19 position of the aglycone, but the glucosyl group at the C19 position of RD did not transfer a positive effect on the solubility of RD, while it does provide RA1G with high solubility (Figure 2c). The solubility of the SGs (mg/mL) in water follows the sequence of RA1G (58.90) > RA (4.55) > RD (0.43) ~ RE (0.34) (empirical solubility equation fitting is shown in Tables S3–S9). The poor solubility and premicellar association of RE and RD even make them unable to form micelles in water. In addition to being solubilized as monomers in water, SGs could also be micellizated via hydrogen bond formation and other molecular interactions, making SGs a biosurfactant in water [6,20]. However, the critical micelle concentrations (*CMC*) of the natural SGs [6,20] are close to their aqueous solubility (Figure 2d, Table S10), which limits their usage as solubilizers or carriers.

Figure 2c also indicates that in ethanol aqueous solution, as the water content increased, the solubility of the four SGs initially climbed and then declined. The highest solubility of the SGs (mg/mL) was achieved in 50% ethanol, with the sequence of RA1G (267.45) > RA (115.42) > RD (4.69) ~ RE (3.79).

In general, hydrogen bond formation between water and solute decides the aqueous solubility. RA1G and RD molecules possess the same number of hydrogen bond donors and accepters, just as RA and RE do. Therefore, the solubility difference between the isomers should come from the difference in the molecular degree of freedom in water.

**Figure 3:**
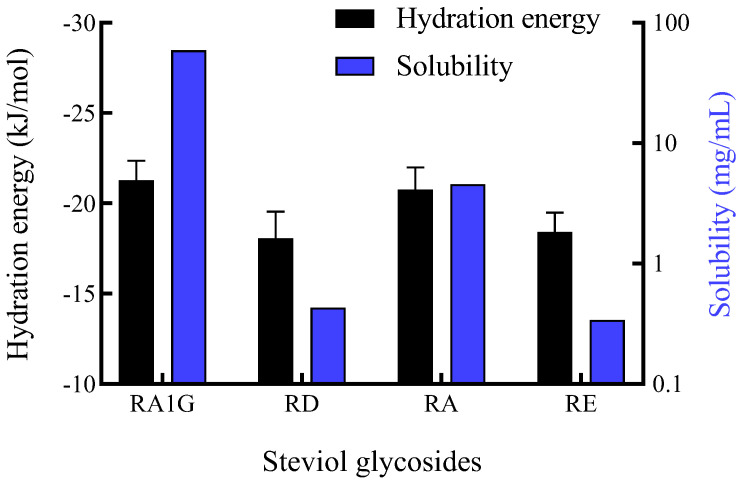
The solvation free energy of SGs and their solubility in water; 25 °C.

As shown in Figure 3, the solvation free energy of SGs in water was consistent with their solubility values. The solvation free energies varied significantly between two pairs of isomers: RA1G (−21.27 kJ/mol) < RD (−18.07 kJ/mol); RA (−20.75 kJ/mol) < RE (−18.41 kJ/mol) (Tables S11–S14). A more negative solvation free energy indicated more energetically favorable interactions between the solute and solvent, implying a thermodynamically favorable dissolution process. It can be inferred that the glycosidic bond is the key determinant of the solubility of SGs. The *β*-1,2 glucosyl moiety at the C19 position in RD and RE did not enhance the solubility of these SGs; instead, it increased the solvation free energy, significantly reducing the solubility of SGs. This effect may stem from the orientation of the hydroxyl groups involved in the bond formation and those external to the steviol ring, which are positioned on the same side. This alignment impedes the efficient formation of hydrogen bonds, thereby affecting solubility (notably resulting in RD having a significantly lower solubility compared to RA). In contrast, the *α*-1,6 glucosyl linkage (which is unique to the RA1G molecule among the four SGs) provides the molecule with greater flexibility in water, thereby enhancing its solubility [28,29]. Introducing a glucosyl moiety connected by an *α*-1,6 bond resulted in lower solvation free energy, thereby raising the solubility of SGs.

Therefore, since the solubility of RA1G in water is 137 times that of its isomer RD, RA1G (*CMC* = 6.25 mg/mL or 5.54 mmol/L) may serve as a better sweet solubilizer for those insoluble additives, such as lipophilic vitamins, such as vitamin D and E.

### 3.3. Crystal Properties, Thermal and pH Stability

The results in Section 3.2 suggest a higher difference in solubility of the SGs in water and in 50% ethanol, which implies a better crystallization potential in alcohol for the SGs, benefiting their purification and application. For example, the solubility of RA1G is 58.90 mg/mL in water and 267.45 mg/mL in 50% ethanol, making its recrystallization easier in alcohol.

**Figure 4:**
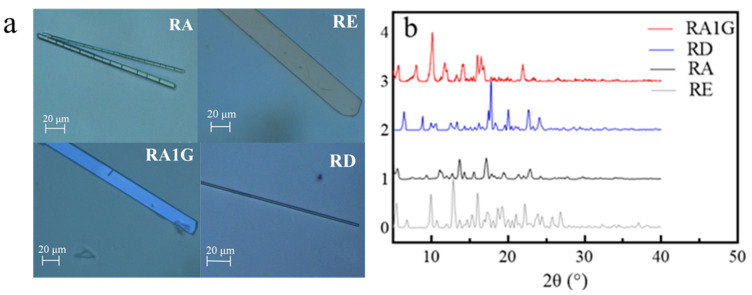
Crystal profile of the isomers. (**a**) Polarizing microscope photo, samples were recrystallized in 80% methanol. (**b**) XRD pattern.

As shown in Figure 4a, the crystals of the isomeric SGs crystallized from 80% methanol are all of sheet structure. The XRD patterns (Figure 4b) illustrate that the C19 substitutes (RD, RE) exhibited more peaks than the C13 substitutes when θ > 25°.

**Figure 5:**
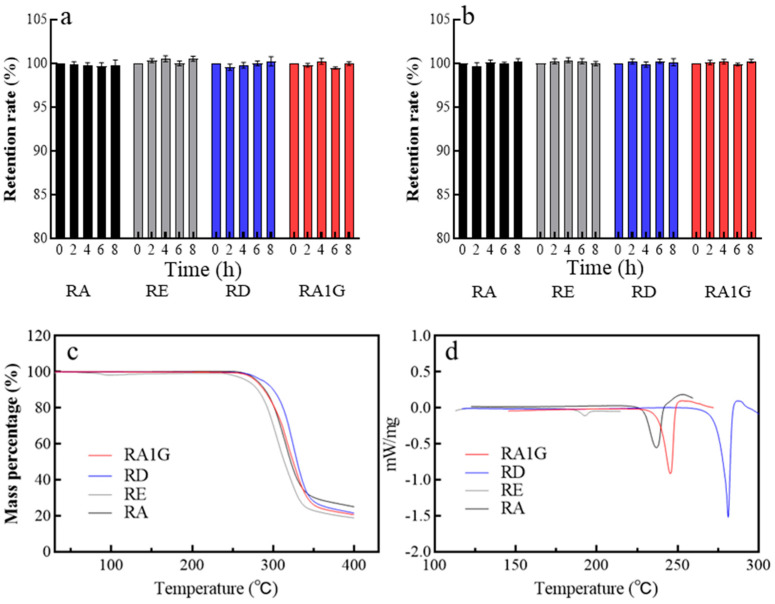
pH and thermal stability of RA, RE, RA1G, and RD. (**a**) pH 1.5, 37 °C. (**b**) pH 6.8, 37 °C. (**c**) TGA profile. (**d**) DSC profile.

As for their stability in a digestive pH environment, the SGs exhibit similar pH stability (Figure 5a,b); they remained stable (at least 98% remained) at pH 1.5 or pH 6.8 in 8 h. The similarity in pH stability among these isomers suggests that the specific linkage type and position of the substituted glycosyl do not significantly influence the overall stability of the molecule in acidic conditions.

The TGA and DSC patterns prove that RA1G is stable until 225 °C (Figure 5c,d). As depicted in Figure 5d, the melting points of the SGs are as follows: 193 °C (RE), 236 °C (RA), 246 °C (RA1G), and 281 °C (RD), compared to literature data, 205–207 °C (RE), 242–244 °C (RA), and 283–286 °C (RD), respectively [30]. Furthermore, the fusion enthalpy (μVs/mg) for these crystals followed the order RE (14.49) < RA1G (203.7) < RA (218.1) < RD (277.7). RE crystals have the lowest melting point and enthalpy of fusion, signifying their relatively weaker thermal stability among the four SG crystals. The results from DSC suggest that the number of sugar moieties positively influences the thermal stability of the steviol glycoside crystals, and *β*-glycosidic bonds exhibit higher stability than *α*-glycosidic bonds.

Taken together, SGs could be used as sugar substitutes in a wide range of food and beverage products without the risk of degradation or loss of sweetness.

### 3.4. Sensory Features of the Isomer Pairs

**Figure 6:**
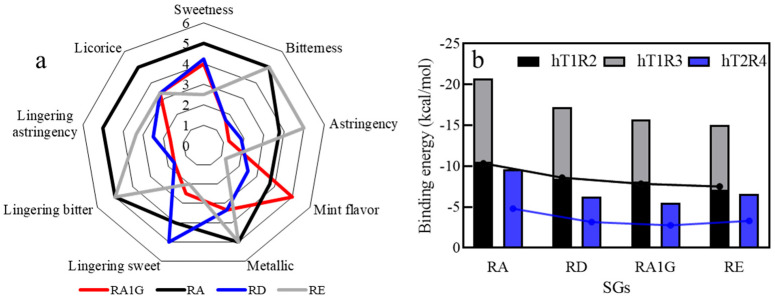
The sensory evaluation of the SGs solution at 25 °C. (**a**) Sensory features; (**b**) binding energy.

Sensory analysis was performed using aqueous solutions sweetened with RA, RE, RD, and RA1G towards nine different taste attributes (Figure 6a). The sweetness data are RA (285–315) > RD ~ RA1G (200–230) > RE (150–170). RA offered the heaviest sweetness and left an astringent aftertaste. RA1G provided a pleasant and pure sweetness, as RD did, plus a mint-like flavor. Therefore, although the glycosylation made RA1G less sweet, it also mitigated the bitterness, making RA1G an ideal sweetener alternating to RA and RD; its milder aftertaste and good solubility endow it with the potential for extensive application in the food industry.

A computational simulation was also conducted to explain the taste difference among the isomers (Figure 6b). Models of sweet taste receptors (hT1R2 and hT1R3) and a bitter taste receptor (hT2R4) were constructed to simulate the mechanism of taste generation when consuming steviol glycosides (Figures S6 and S7, Tables S15–S17). Through molecular docking, for sweetness, the cumulative binding energy between SGs and sweet receptors (hT1R2 and hT1R3) was determined as follows: RA (−20.7 kcal/mol) < RD (−17.2 kcal/mol) < RA1G (−15.7 kcal/mol) < RE (−15 kcal/mol). Among the four steviol glycosides, RA possesses the highest sweetness. Introducing a glycosyl group at the C19 position can slightly reduce the sweetness.

Bitterness was assessed through the interaction with the bitter taste receptor hT2R4. RA showed the highest binding energy at −9.6 kcal/mol, suggesting it also has the highest bitter taste perception among the SGs. The binding energy between SGs and bitter taste receptor hT2R4 was followed by RE at −6.6 kcal/mol, RA1G at −6.3 kcal/mol, and RD, which had the lowest binding energy at −5.5 kcal/mol, indicating the least bitter perception. The binding energy between the receptor and SG exhibits a correlation with the intensity of sweetness and bitterness (Figure 6b). Introducing a glucosyl group at the C19 position can enhance the taste quality of steviol glycosides. However, the type of glycosidic bond (*β*-1,2 and *α*-1,6 glycosidic bonds) has a negligible effect on taste quality, with RD and RA1G demonstrating comparable sweetness and bitterness.

In another hand, the variation in sweetness could be attributed to the hydrogen bonds between SG and the receptor [31], where the quantity of hydrogen bonds influences the magnitude of the binding energy (Figures S8 and S9, Table S18). However, within the bitter taste receptors, the binding of SGs to the protein appears to be more complex, with no direct correlation between hydrogen bonds and the magnitude of binding energy (Figure S10, Table S19). This suggests that other types of non-covalent interactions, such as hydrophobic interactions and van der Waals forces, may collectively contribute to the bitterness.

## 4. Conclusions

A new *α*-1,6-glycosidic isomer of rebaudioside D (RD), namely RA1G, was synthesized with the glucosylation of rebaudioside A (RA) and sucrose, catalyzed by an alternansucrase. The RA1G yield can reach 41% in 10 h when the reaction is conducted at 32 °C with 10 mg/mL RA, an RA-to-sucrose ratio of 1:5.6, and an enzyme dosage of 8 U/g RA.

The RA1G possesses similar sweetening properties to RD, with a solubility 137 times greater than that of RD and 13 times that of RA. The calculated solvation free energy outcomes highlight the significant impact of glycosidic bond types on the solubility of steviol glycosides, which is RA1G (−21.27 ± 1.08 kJ/mol) < RA (−20.75 ± 1.24 kJ/mol) < RE (−18.41 ± 1.08 kJ/mol) ~ RD (−18.07 ± 1.47 kJ/mol), corresponding to their experimental solubility values: RA1G (58.90 mg/mL) > RA (4.55 mg/mL) > RD (0.43 mg/mL) ~ RE (0.34 mg/mL). The *β*-1,2 glucosyl group at the C19 position increases the solvation free energy, thereby significantly diminishing the solubility of SGs. Conversely, the *α*-1,6 glucosyl linkage decreases the solvation free energy, granting the molecule enhanced flexibility in water and thereby improving its solubility. Although the introduction of hydrophilic glycosyl groups does not invariably lead to an improvement in solubility, it shows a positive influence on both polarity and thermal stability.

Furthermore, in this study, with homology modeling and molecular docking, the potential binding sites of SGs with sweet and bitter taste receptors were elucidated. Within constructed taste receptor models, an inverse relationship was observed between binding energy and the intensity of sweetness or bitterness. The introduction of a glucosyl moiety at the C19 position was observed to diminish both bitterness and sweetness while introducing a glucosyl moiety at the C13 position increased sweetness and reduced bitterness. However, the type of glycosidic bond has a minor impact on the taste profile. These differences could be attributed to the varying spatial hindrance affecting the binding with taste receptors.

By integrating theoretical simulations with calculations, we elucidated the reasons behind the varying physicochemical properties of steviol glycoside isomers, clarifying the impact of glycosidic linkage types and positions of the substituted glycosyl on their physicochemical properties. This can provide valuable guidance for the synthesis and development of innovative sweeteners.

Taken together, the results indicate that the glucosyl linkage type and position have a stronger impact on the properties of the SGs than the number of glucosyl moieties. The SGs isomeric pairs could be used as sugar substitutes in a wide range of food and beverage products without the risk of degradation or loss of sweetness.

## Data Availability

The original contributions presented in the study are included in the article/Supplementary Materials; further inquiries can be directed to the corresponding author.

## Supplementary Materials

The following supporting information can be downloaded at https://www.mdpi.com/article/10.3390/foods14111917/s1: Figure S1. Three sugar residues from the RA1G identified with methylation analysis (a) total ion current; Figure S2. 1H NMR of RA1G. a: Full spectrum; b: the anomeric region of the sugar units; c: aglycone group; Figure S3.13C NMR of RA1G; Figure S4. 2D NMR profiles of the monoglucosyl-substituted rebaudioside A. (a) COSY spectra. a1, Full spectrum; a2, enlarged spectrum obtained for the anomeric region of the sugar units; a3, enlarged proton spectrum obtained for the aglycone group. (b) TOCSY spectra. b1, Full spectrum; b2, an enlarged anomeric spectrum region for the sugar units; b3, an enlarged proton spectrum for the aglycone group. (c) Enlarge HSQC spectrum. c1: sugar units; c2: aglycone group. (d) HMBC spectra of the monoglucosyl-substituted rebaudioside A. (e) Structure of the RA1G; Figure S5. Changes in sucrose, glucose, fructose content of transglycosides and hydrolysis. 32 °C, 4 U/g sucrose, solid line: RA: sucrose (mol/mol) = 1: 5.6, 10 mgRA/mL. Dotted line: without RA, 20 mg sucrose/mL; Figure S6. The homology models of hT1R2 (a) and hT1R3 (b), and Ramachandran plot of hT1R2 (c) and hT1R3 (d); Figure S7. The models and Ramachandran plot of hT2R4; Figure S8. The interaction patterns of four steviol glycosides with hT1R2. Yellow dash: Hydrogen bonds; Figure S9. The interaction patterns of four steviol glycosides with hT1R3. Yellow dash: Hydrogen bonds; Figure S10. The interaction patterns of four steviol glycosides with hT2R4. Yellow dash: Hydrogen bonds; Table S1. Linking mode of methylation analysis glucose residues; Table S2. Apelblat equation calculates the solubility parameters of steviol glycosides in ethanol; Table S3. Polynomial empirical equations calculate the solubility parameters of steviol glycosides in ethanol; Table S4. λh equation to calculate the solubility parameters of steviol glycosides in ethanol; Table S5. Fitting results of three equations (ethanol system); Table S6. Apelblat equation calculates the solubility parameters of steviol glycosides in 95% ethanol; Table S7. Polynomial empirical equations calculate the solubility parameters of steviol glycosides in 95% ethanol; Table S8. λh equation to calculate the solubility parameters of steviol glycosides in 95% ethanol; Table S9. Fitting results of three equations (95% ethanol); Table S10. Surface activity of the steviol glycosides; Table S11. The free energy of RE decoupling process in water; Table S12. The free energy of RA decoupling process in water; Table S13. The free energy of RD decoupling process in water; Table S14. The free energy of RA1G decoupling process in water; Table S15. The suitable protein templates for hT1R2 and hT1R3; Table S16. The valuation of the models of hT1R2, hT1R3, and hT2R4; Table S17. The affinity of four steviol glycosides with hT1R2, hT1R3, and hT2R4; Table S18. The key amino acids in sweet receptors interacted with SGs; Table S19. The key amino acids in bitter receptors interacted with SGs. References [32,33] are cited in Supplementary Materials.

## Abbreviations

The following abbreviations are used in this manuscript:

ARD	Average relative deviation
CMC	Critical micelle concentrations
DSC	Differential scanning calorimetry
NVT	Constant number of particles, volume, and temperature ensemble
NPT	Constant number of particles, pressure, and temperature ensemble
RA	Rebaudioside A
RE	Rebaudioside E
RD	Rebaudioside D
RA1G	Mono-glucosyl RA
RMSD	Root mean square deviation
SG	Steviol glycoside
St	Stevioside
TGA	Thermogravimetric analysis
T/K	Temperature in Kelvin
VFTD	Venus flytrap domain
XRD	X-ray diffraction
