# Supplementary material for: Structure–Property Relevance of Two Pairs of Isomeric Steviol Rebaudiosides and the Underlying Mechanism
Source: Foods. 2025 May 28;14(11):1917. doi: 10.3390/foods14111917 (PMC12155493; doi:10.3390/foods14111917)
Supplement: Supplementary file 1 [file foods-14-01917-s001.zip › foods-3646531-supplementary.pdf]

Supporting material

Structure-property relevance of two pairs of isomeric steviol rebaudiosides and the underlying mechanism

Zhuoyu Zhou [1,+], Wanjie Wang [1,+], Qinbing Guo [2], Haijun Wang [1], Yongmei Xia [1,*]

The experiment methods used in this Supporting Material are mainly listed in the methods section in main text, except the computational method stated specifically in the modeling section here.

The following supporting information can be downloaded at: https://www.mdpi.com/article/doi/s1.

Figure S1. Three sugar residues from the RA1G identified with methylation analysis (a) total ion current.

Figure S2. 1H NMR of RA1G. a: Full spectrum; b: the anomeric region of the sugar units; c: aglycone group.

Figure S3.13C NMR of RA1G.

Figure S4. 2D NMR profiles of the monoglucosyl-substituted rebaudioside A. (a) COSY spectra. a1, Full spectrum; a2, enlarged spectrum obtained for the anomeric region of the sugar units; a3, enlarged proton spectrum obtained for the aglycone group. (b) TOCSY spectra. b1, Full spectrum; b2, an enlarged anomeric spectrum region for the sugar units; b3, an enlarged proton spectrum for the aglycone group. (c) Enlarge HSQC spectrum. c1: sugar units; c2: aglycone group. (d) HMBC spectra of the monoglucosyl-substituted rebaudioside A. (e) Structure of the RA1G.

Figure S5. Changes in sucrose, glucose, fructose content of transglycosides and hydrolysis. 32 ° C, 4U / g sucrose, solid line: RA: sucrose (mol / mol) = 1: 5.6, 10 mgRA / mL. Dotted line: without RA, 20 mg sucrose / mL.

Figure S6. The homology models of hT1R2 (a) and hT1R3 (b), and Ramachandran plot of hT1R2 (c) and hT1R3 (d).

Figure S7. The models and Ramachandran plot of hT2R4.

Figure S8. The interaction patterns of four steviol glycosides with hT1R2. Yellow dash: Hydrogen bonds.

Figure S9. The interaction patterns of four steviol glycosides with hT1R3. Yellow dash: Hydrogen bonds.

Figure S10. The interaction patterns of four steviol glycosides with hT2R4. Yellow dash: Hydrogen bonds. Table S1. Linking mode of methylation analysis glucose residues.

Table S2. Apelblat equation calculates the solubility parameters of steviol glycosides in ethanol.

Table S3. Polynomial empirical equations calculate the solubility parameters of steviol glycosides in ethanol.

Table S4. λh equation to calculate the solubility parameters of steviol glycosides in ethanol.

Table S5. Fitting results of three equations (ethanol system).

Table S6. Apelblat equation calculates the solubility parameters of steviol glycosides in 95% ethanol.

Table S7. Polynomial empirical equations calculate the solubility parameters of steviol glycosides in 95% ethanol.

Table S8. λh equation to calculate the solubility parameters of steviol glycosides in 95% ethanol.

Table S9. Fitting results of three equations (95% ethanol).

Table S10. Surface activity of the steviol glycosides.

Table S11. The free energy of RE decoupling process in water.

Table S12. The free energy of RA decoupling process in water.

Table S13. The free energy of RD decoupling process in water.

Table S14. The free energy of RA1G decoupling process in water.

Table S15. The suitable protein templates for hT1R2 and hT1R3.

Table S16. The valuation of the models of hT1R2, hT1R3 and hT2R4.

Table S17. The affinity of four steviol glycosides with hT1R2, hT1R3 and hT2R4.

Table S18. The key amino acids in sweet receptors interacted with SGs.

Table S19. The key amino acids in bitter receptors interacted with SGs.

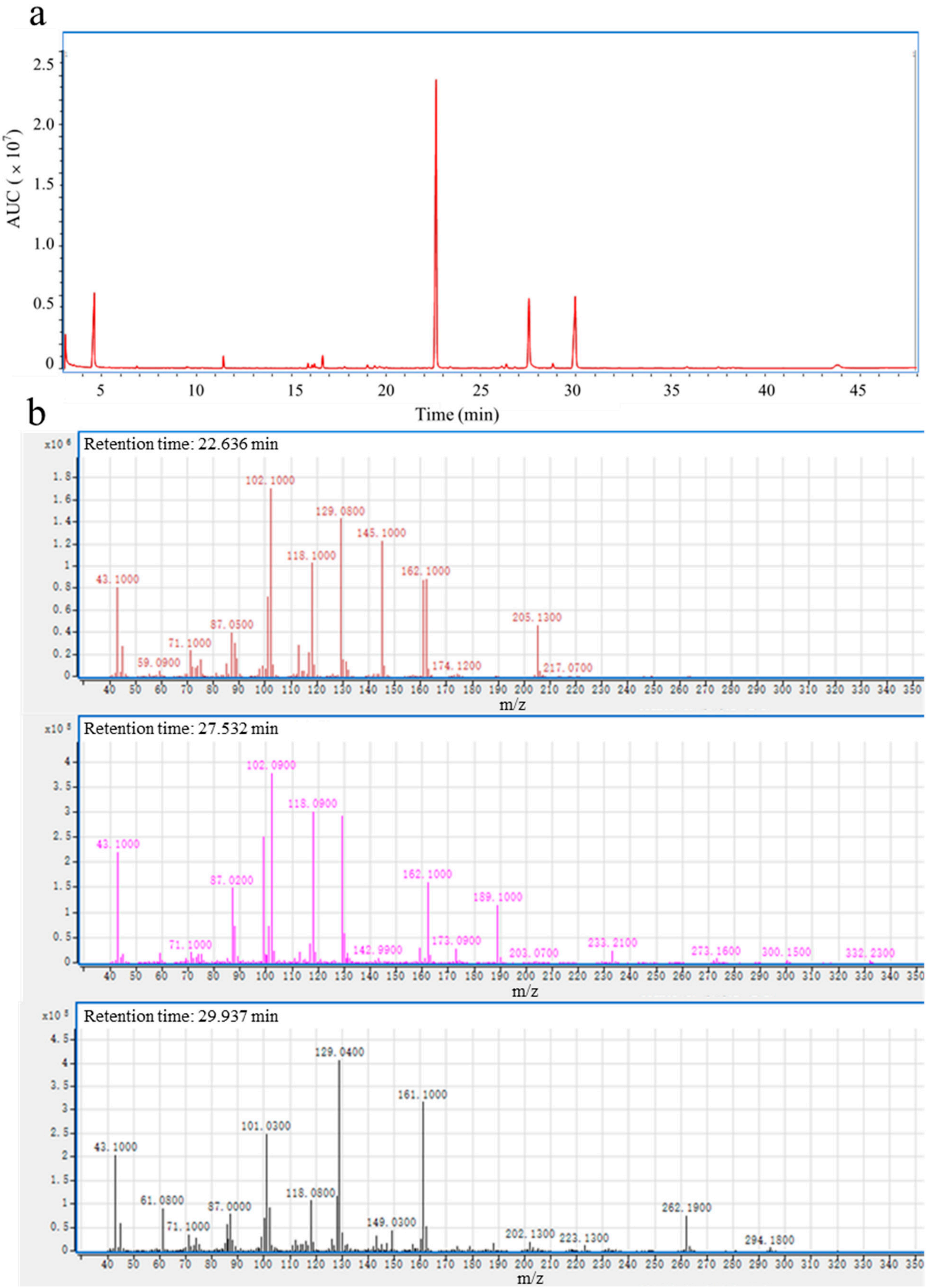

Figure S1. Three sugar residues from the RA1G identified with methylation analysis (a) total ion current

chromatogram of the reaction mixture. (b) mass spectra of molecules with retention times of 22.636 min, 27.532 min, and 29.937 min, respectively

Table S1. Linking mode of methylation analysis glucose residues

Retention Time (min)	Types of linkage	Mol ratio (%)
22.636	T-Glc*P*	2.97
27.532	6-Glc*P*	0.75
29.937	2,3-Glc*P*	1.00

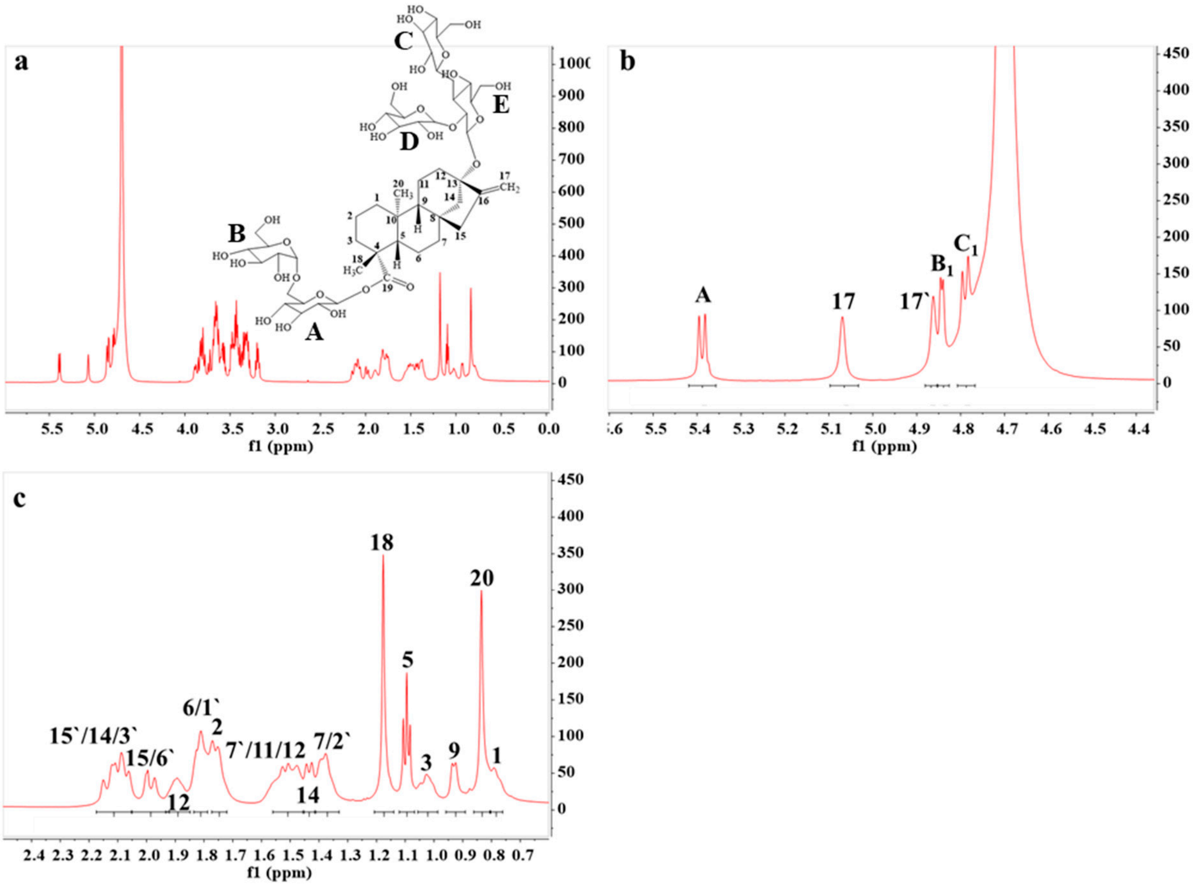

Figure S2. ^{1}H NMR of RA1G. a: Full spectrum. b: the anomeric region of the sugar units. c: aglycone group

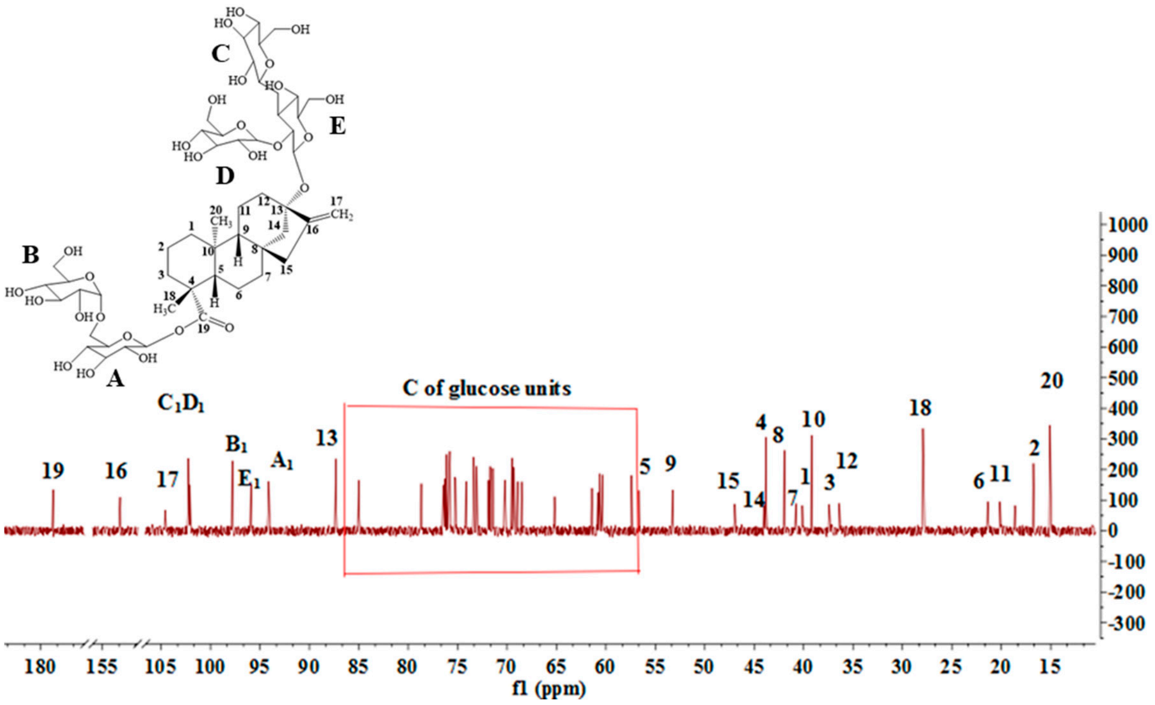

Figure S3. ^{13}C NMR of RA1G

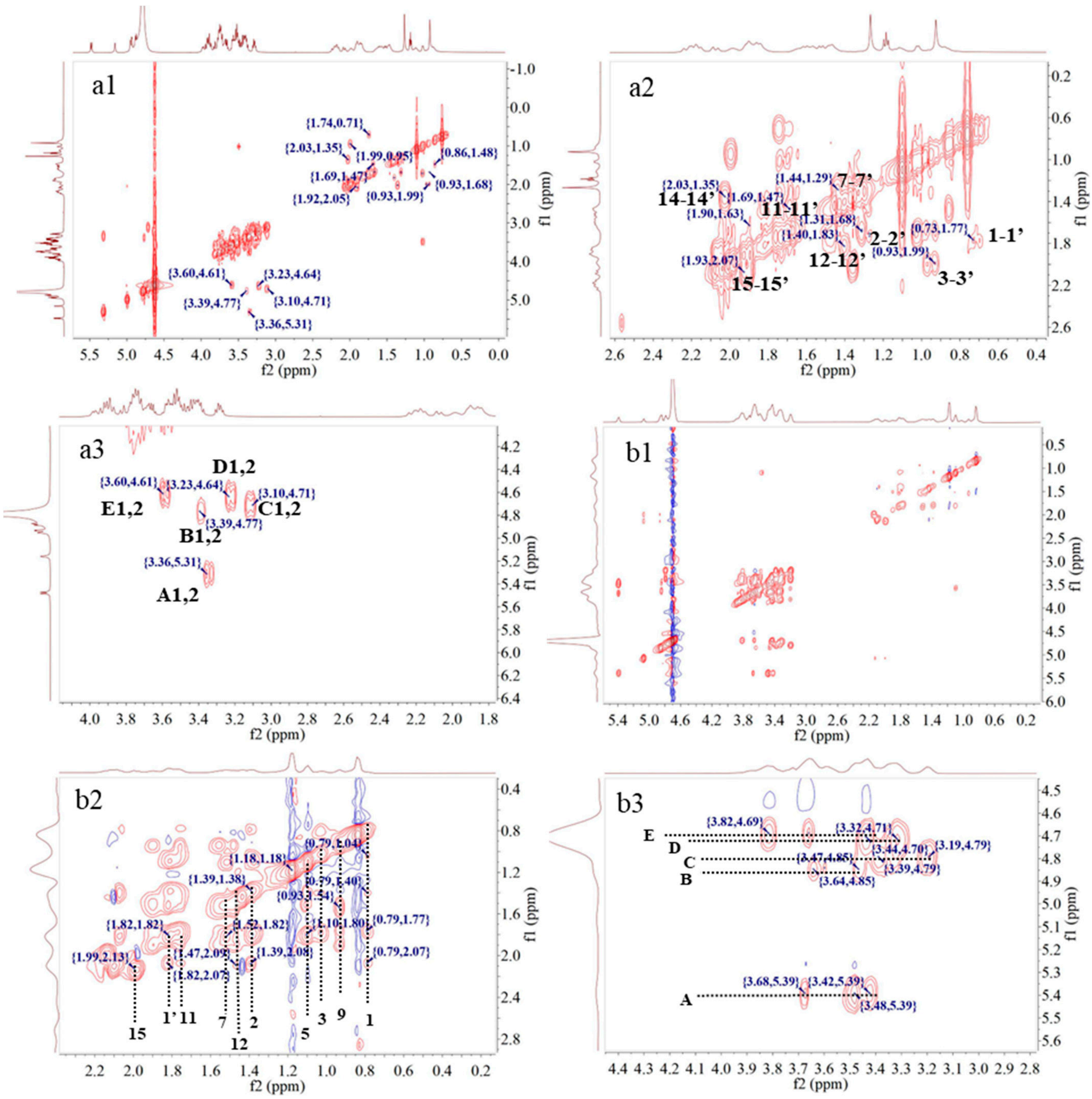

a1
{1.74,0.71}
{2.03,1.35}
{1.99,0.95}
{0.86,1.48}
{1.69,1.47}
{1.92,2.05}
{0.93,1.99}
{0.93,1.68}
{3.60,4.61}
{3.23,4.64}
{3.39,4.77}
{3.10,4.71}
{3.36,5.31}
f2 (ppm)
f1 (ppm)
a2
{2.03,1.35}
14-14'
{1.69,1.47}
11-11'
{1.44,1.29}
7-7'
{1.90,1.63}
{1.31,1.68}
2-2'
{0.73,1.77}
1-1'
{1.40,1.83}
12-12'
{0.93,1.99}
3-3'
{1.93,2.07}
15-15'
f2 (ppm)
f1 (ppm)
a3
D1,2
{3.60,4.61}
{3.23,4.64}
{3.10,4.71}
E1,2
C1,2
{3.39,4.77}
B1,2
{3.36,5.31}
A1,2
f2 (ppm)
f1 (ppm)
b1
f2 (ppm)
f1 (ppm)
b2
{1.18,1.18}
{0.79,1.04}
{1.39,1.38}
{0.79,1.40}
{0.93,1.54}
{1.82,1.82}
{1.52,1.82}
{1.10,1.80}
{0.79,1.77}
{1.99,2.13}
{1.47,2.09}
{1.39,2.08}
{0.79,2.07}
{1.82,2.07}
15
1'
11
7
12
2
5
3
9
1
f2 (ppm)
f1 (ppm)
b3
{3.82,4.69}
{3.32,4.71}
E
D
{3.44,4.70}
{3.19,4.79}
C
{3.47,4.85}
{3.39,4.79}
B
{3.64,4.85}
{3.68,5.39}
{3.42,5.39}
A
{3.48,5.39}
f2 (ppm)
f1 (ppm)

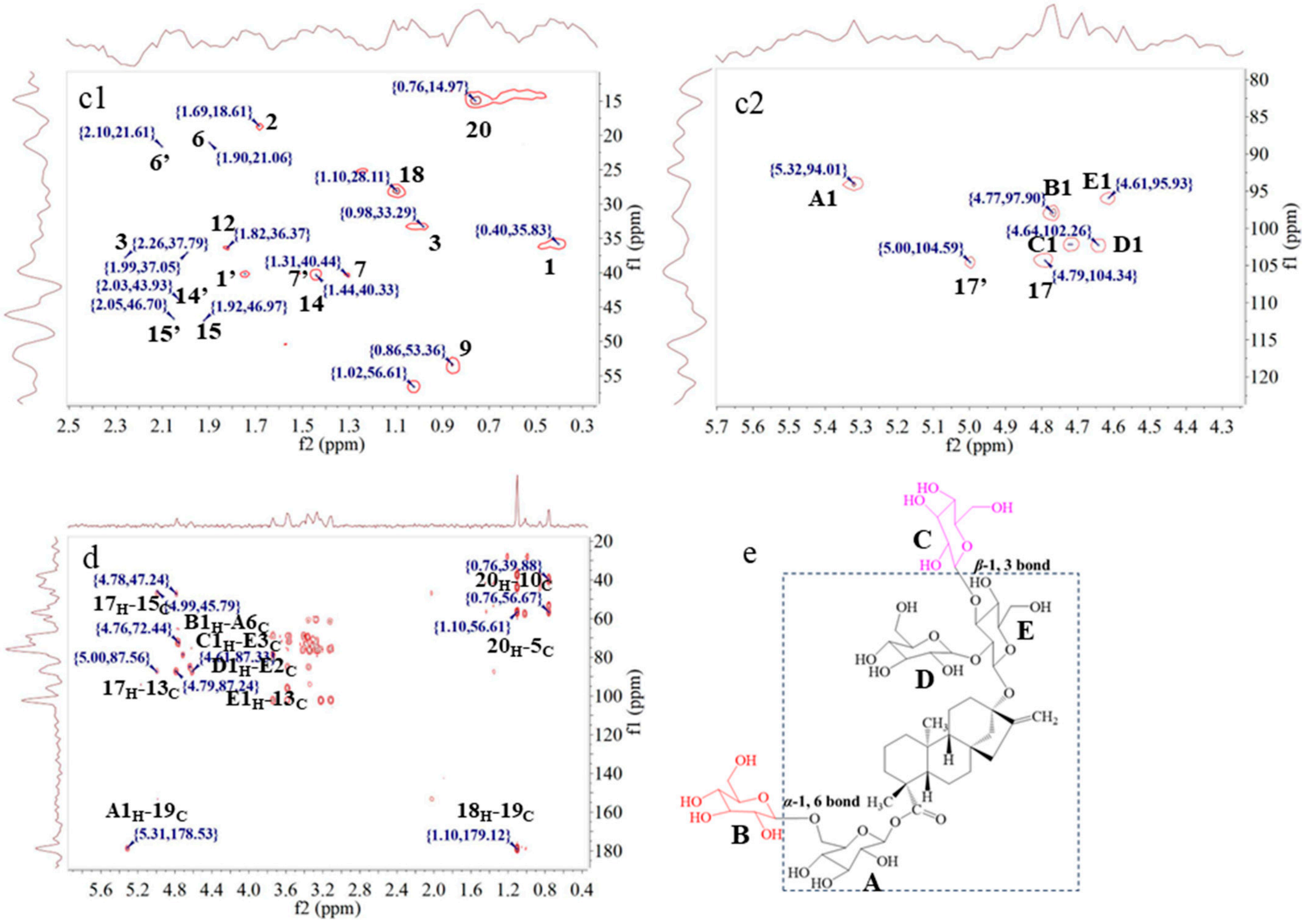

Figure S4. 2D NMR profiles of the monoglucosyl-substituted rebaudioside A. (**a**) COSY spectra. a1, Full spectrum. a2, enlarged spectrum obtained for the anomeric region of the sugar units. a3, enlarged proton spectrum obtained for the aglycone group. (**b**) TOCSY spectra. b1, Full spectrum. b2, an enlarged anomeric spectrum region for the sugar units. b3, an enlarged proton spectrum for the aglycone group. (**c**) Enlarge HSQC spectrum. c1: sugar units. c2: aglycone group. (**d**) HMBC spectra of the monoglucosyl-substituted rebaudioside A. (**e**) Structure of the RA1G.

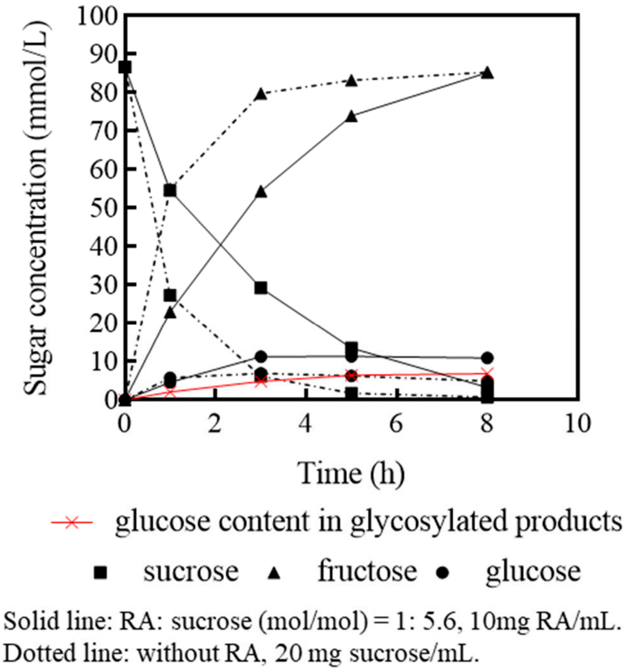

Figure S5. Changes in sucrose, glucose, fructose content of transglycosides and hydrolysis. 32℃, 4U / g sucrose, solid line: RA: sucrose (mol / mol) = 1: 5.6, 10 mgRA / mL. Dotted line: without RA, 20 mg sucrose / mL.

Fitting the solubility of four steviol glycosides using empirical formulas

Table S2. Apelblat equation calculates the solubility parameters of steviol glycosides in ethanol.

	A	B	C	10^6 RMSD	ARD
RE	-1.56×10^3	6.79×10^4	2.32×10^2	7.31	0.030
RA	-5.11×10^2	2.30×10^4	7.43×10	0.77	0.015
RD	1.38×10	-1.40×10^3	-3.60	0.36	0.023
RA1G	-3.11×10^2	1.25×10^4	4.53×10	0.95	0.018

Table S3. Polynomial empirical equations calculate the solubility parameters of steviol glycosides in ethanol.

	A	B	C	D	10^6 RMSD	ARD
RE	-2.04×10^{-1}	2.01×10^{-3}	-6.61×10^{-6}	7.24×10^{-9}	2.47	0.016
RA	7.37×10^{-3}	-6.45×10^{-5}	1.87×10^{-7}	-1.79×10^{-10}	0.75	0.015
RD	3.25×10^{-3}	-3.08×10^{-5}	9.73×10^{-8}	-1.02×10^{-10}	0.32	0.026
RA1G	-2.07×10^{-2}	1.99×10^{-4}	-6.39×10^{-7}	6.85×10^{-10}	0.48	0.009

Table S4. λh equation to calculate the solubility parameters of steviol glycosides in ethanol

	λ	h	10^5 RMSD	ARD
RE	1.45×10^{-1}	5.19×10^4	1.34	0.185
RA	-2.09×10^{-5}	2.50×10^7	0.24	0.049
RD	-9.30×10^{-6}	1.51×10^8	0.03	0.025
RA1G	2.96×10^4	5.82×10^6	0.17	0.038

Table S5. Fitting results of three equations (ethanol system)

T/K	10^5 x_1^{exp}	10^5 x_1^{cal}		
		Apelblat	Polynomial Empirical Equation	λh
		RE		
298.15	3.92	3.99	3.94	1.87
303.15	4.56	4.40	4.58	2.81
313.15	6.23	6.34	6.03	6.81
323.15	10.96	11.24	11.34	12.76
333.15	25.22	23.87	24.83	25.40
338.15	35.84	36.96	36.02	35.32
		RA		
298.15	4.41	4.33	4.36	4.04
303.15	4.06	4.17	4.15	4.15

313.15	4.12	4.12	4.09	4.40
323.15	4.48	4.38	4.41	4.67
333.15	4.89	4.97	5.01	4.98
338.15	5.44	5.42	5.39	5.15
		RD		
298.15	1.04	1.04	1.06	1.05
303.15	1.07	1.06	1.04	1.06
313.15	1.03	1.09	1.08	1.08
323.15	1.18	1.12	1.14	1.11
333.15	1.13	1.14	1.16	1.14
338.15	1.15	1.15	1.14	1.16
		RA1G		
298.15	2.86	2.91	2.85	2.74
303.15	3.15	3.10	3.18	3.05
313.15	3.65	3.61	3.67	3.73
323.15	4.31	4.36	4.25	4.53
333.15	5.27	5.42	5.35	5.46
338.15	6.26	6.11	6.22	5.98

Table S6. Apelblat equation calculates the solubility parameters of steviol glycosides in 95% ethanol.

	A	B	C	10^5 RMSD	ARD
RE	-8.80×10^2	3.46×10^4	1.32×10^2	0.83	0.050
RA	-4.69×10^2	2.06×10^4	6.87×10	1.13	0.035
RD	-1.03×10^3	4.49×10^4	1.52×10^2	0.02	0.035
RA1G	-2.51×10^2	1.13×10^4	3.73×10	2.69	0.062

Table S7. Polynomial empirical equations calculate the solubility parameters of steviol glycosides in 95% ethanol.

	A	B	C	D	10^5 RMSD	ARD
RE	-0.48	4.62×10^{-3}	-1.50×10^{-5}	1.61×10^{-8}	0.75	0.126
RA	-0.24	2.34×10^{-3}	-7.46×10^{-6}	7.95×10^{-9}	0.32	0.009
RD	-0.007	6.79×10^{-5}	-2.19×10^{-7}	2.36×10^{-10}	0.01	0.026
RA1G	-0.44	4.32×10^{-3}	-1.42×10^{-5}	1.55×10^{-8}	1.09	0.046

Table S8. λh equation to calculate the solubility parameters of steviol glycosides in 95% ethanol

	λ	h	10^5 RMSD	ARD
RE	9.05×10^{-1}	1.02×10^4	1.37	0.215
RA	5.04×10^{-5}	1.69×10^5	1.95	0.057

RD	8.85×10^{-4}	4.43×10^{6}	3.54	1.503
RA1G	3.43	2.42×10^{3}	3.60	0.270

Table S9. Fitting results of three equations (95% ethanol)

T/K	$10^5 x_1^{exp}$	$10^5 x_1^{cal}$		
		Apelblat	Polynomial Empirical Equation	λh
		RE		
298.15	2.68	2.66	2.04	1.28
303.15	3.367	3.54	4.63	2.13
313.15	7.62	6.78	6.79	5.66
323.15	12.97	14.22	12.93	14.12
333.15	31.99	32.24	32.70	33.34
338.15	51.18	49.82	50.76	50.26
		RA		
298.15	23.00	23.78	22.85	22.00
303.15	24.75	23.90	24.99	23.57
313.15	26.29	25.49	26.32	26.98
323.15	28.11	28.93	27.70	30.81
333.15	33.34	34.83	33.90	35.13
338.15	40.57	38.93	40.29	37.50
		RD		
298.15	0.27	0.28	0.27	0.20
303.15	0.29	0.29	0.31	0.25
313.15	0.38	0.36	0.36	0.38
323.15	0.46	0.49	0.47	0.56
333.15	0.77	0.78	0.77	0.81
338.15	1.04	1.01	1.04	0.97
		RA1G		
298.15	7.99	8.32	8.22	2.39
303.15	8.54	7.93	8.32	3.78
313.15	9.46	9.69	8.82	9.08
323.15	15.88	16.93	17.49	20.70
333.15	45.40	40.42	43.58	44.74
338.15	65.25	69.39	66.11	64.72

Table S10. Surface activity of the steviol glycosides

SGs	CMC (mmol/L)	γ_{cmc} mN/m	$\prod_{cmc}$ mN/m	Γ_{max} nmol/cm^2	A_{min} (Å)	$\triangle G_m^\circ$ kJ/mol	$\triangle G_{ads}^\circ$ kJ/mol
RA	4.94	54.27	17.93	0.149	111.92	-23.13	-35.16
RA1G	5.54	54.33	17.87	0.152	108.93	-22.84	-34.60
RE	\	\	\	\	\	\	\
RD	\	\	\	\	\	\	\

Table S11. The free energy of RE decoupling process in water

Point	△G (kJ/mol)	Point	△G (kJ/mol)
0-1	10.10 ± 0.04	10-11	6.47 ± 0.01
1-2	10.08 ± 0.04	11-12	5.35 ± 0.06
2-3	9.88 ± 0.03	12-13	3.70 ± 0.05
3-4	9.64 ± 0.04	13-14	1.45 ± 0.11
4-5	9.37 ± 0.06	14-15	-2.60 ± 0.22
5-6	9.17 ± 0.08	15-16	-14.00 ± 0.32
6-7	8.89 ± 0.03	16-17	-28.70 ± 0.25
7-8	8.45 ± 0.04	17-18	-24.81 ± 0.16
8-9	7.96 ± 0.01	18-19	-14.70 ± 0.04
9-10	7.37 ± 0.06	19-20	-4.66 ± 0.04

$\triangle G_{decoupling}$ =18.41±0.45 kJ/mol

$\triangle G_{solvation}$ = -18.41±0.45 kJ/mol

Table S12. The free energy of RA decoupling process in water

Point	△G (kJ/mol)	Point	△G (kJ/mol)
0-1	10.35±0.06	10-11	5.47±0.04
1-2	10.23±0.11	11-12	3.95±0.08
2-3	9.93±0.08	12-13	1.76±0.09
3-4	9.59±0.06	13-14	-2.34±0.26
4-5	9.45±0.08	14-15	-12.89±0.97
5-6	9.4±0.08	15-16	-29.25±0.36
6-7	9±0.07	16-17	-24.79±0.05
7-8	8.45±0.06	17-18	-14.76±0.07
8-9	7.96±0.05	18-19	-4.67±0.01
9-10	7.36±0.06	19-20	5.47±0.04

$\triangle G_{decoupling}$ =20.75±1.24 kJ/mol

$\triangle G_{solvation =}$ -20.75±1.24 kJ /mol

Table S13. The free energy of RD decoupling process in water

Point	△G (kJ/mol)	Point	△G (kJ/mol)
0-1	11.7±0.01	10-11	7.6±0.06
1-2	11.6±0.01	11-12	6.4±0.05
2-3	11.12±0.04	12-13	4.69±0.04
3-4	10.65±0.1	13-14	2.06±0.06

4-5	10.48±0.07	14-15	-2.97±0.21
5-6	10.24±0.05	15-16	-15.53±0.92
6-7	9.85±0.02	16-17	-34.92±0.47
7-8	9.49±0.01	17-18	-29.17±0.09
8-9	9.2±0.04	18-19	-17.31±0.08
9-10	8.42±0.01	19-20	-5.52±0.03

$\triangle G_{decoupling}$ =18.07±1.47 kJ/mol

$\triangle G_{solvation}$ = - 18.07±1.47 kJ /mol

Table S14. The free energy of RA1G decoupling process in water

Point	△G (kJ/mol)	Point	△G (kJ/mol)
0-1	11.46±0.16	10-11	7.51±0.07
1-2	11.59±0.16	11-12	6.33±0.09
2-3	11.53±0.18	12-13	4.59±0.06
3-4	10.91±0.13	13-14	2.40±0.08
4-5	10.57±0.09	14-15	-1.03±0.09
5-6	10.26±0.02	15-16	-15.56±0.26
6-7	9.90±0.02	16-17	-34.71±0.25
7-8	9.53±0.06	17-18	-28.93±0.09
8-9	9.07±0.04	18-19	-17.20±0.05
9-10	8.44±0.06	19-20	-5.39±0.03

△G decoupling =21.27±1.08 kJ/mol

△G solvation = - 21.27±1.08 kJ/mol

Homology Modeling of Sweet Taste Receptors hT1R2, hT1R3

It is now generally acknowledged that the majority of sweeteners interact with the Venus Flytrap Domain (VFTD) of the sweet taste receptor. This interaction prompts the VFTD's two lobes to close, subsequently initiating a cascade of conformational changes within the Cysteine-rich Domain (CRD), Transmembrane Domain (TMD), and Intracellular Domain (ID). These alterations collectively transition the receptor from a resting to an active state(Kim, Chen, Abrol, Goddard, & Guthrie, 2017).

The sweet taste receptor belongs to the class C GPCR family, characterized by large extracellular domains. Its Venus Flytrap domain (VFTD) shares structural similarities with the calcium-sensing receptor. Therefore, homology modeling has utilized templates from the medaka fish (Oryzias latipes) sweet taste receptor (PDB ID: 5X2M), the calcium-sensing receptor (PDB ID: 7E6T), and the class C GPCR metabotropic glutamate receptor (PDB ID: 5CGC).

The amino acid sequences for human T1R2 (NP_689418.2) and T1R3 (NP_689414.1) were retrieved from the NCBI database. A BLAST search identified the most closely related templates to the sweet taste receptors, categorized into three groups: fish sweet taste receptors, class C GPCR metabotropic glutamate receptors, and human calcium-sensing receptors. The templates exhibiting the highest sequence similarity were selected for homology modeling using Modeller 10.4. These include the medaka fish taste receptor (PDB ID: 5X2M), the class C GPCR metabotropic glutamate receptor (PDB ID: 5CGC), and the human calcium-sensing receptor (PDB ID: 7E6T).

Briefly, we performed homology modeling of human sweet taste receptors hT1R2 and hT1R3(Kim et al., 2017; Zhou, Li, Wang, & Xia, 2024)using Discovery Studio 2.5, based on the crystal structure of the medaka fish taste receptor (PDB ID: 5X2M). For the bitter taste receptor hT2R4, modeling was performed via the I-TASSER web server. Molecular docking between SGs and the taste receptors was then conducted using AutoDock Vina 1.5.7. The calculated binding affinities were used to evaluate the binding capabilities of the sweeteners to their binded receptors, respectively.

Table S15. The suitable protein templates for hT1R2 and hT1R3

	hT1R2				hT1R3			
	Score	E value	Query Cover	Percent identity	Score	E value	Query Cover	Percent identity
5X2M	253	1e-74	55%	33.89%	322	2e-100	55%	37.29%
5CGC	46.2	2e-04	9%	34.18%	41.2	0.008	9%	29.63%
7E6T	387	6e-121	94%	31.36%	394	1e-123	94%	30.12%

The sequences of hT1R2 and hT1R3 were aligned with the sequences of the chosen templates. Following the alignment, Modeller 10.4 was then utilized to perform homology modeling, resulting in the construction of ten models for each protein sequence. The best model was selected based on the lowest DOPE score. The final model was validated using Ramachandran plots and Verify 3D to ensure structural reliability.

Homology Modeling:

Table S16. The valuation of the models of hT1R2, hT1R3 and hT2R4

	Verify 3D (%)	Ramachandran Plot			
		Core (%)	Allowed (%)	Generously Allowed (%)	Disallowed (%)
hT1R2	34.09	81.4	13.5	2.9	2.1
hT1R3	47.18	84.8	11.1	1.9	2.2
hT2R4	18.06	87.5	11.1	1.4	0.0

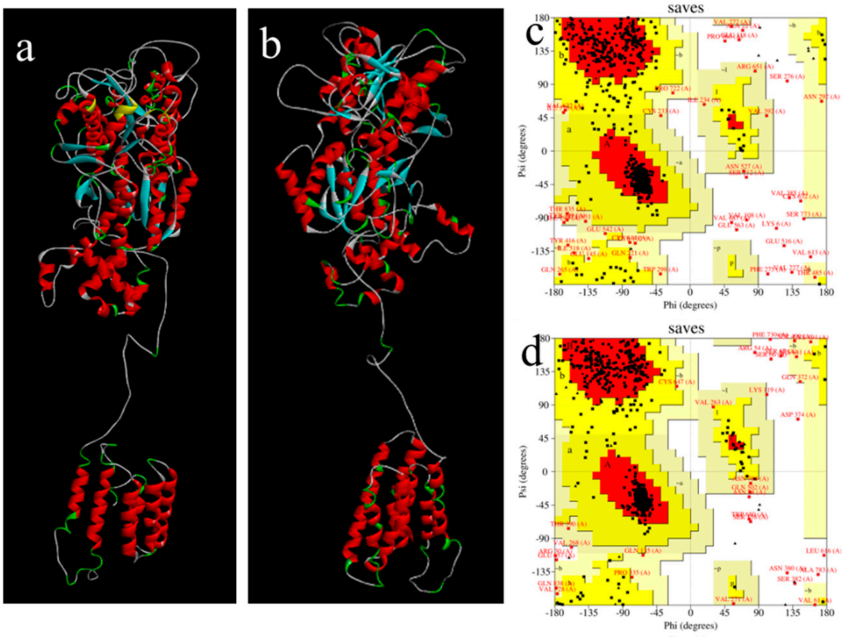

Figure S6. The homology models of hT1R2 (a) and hT1R3 (b), Ramachandran plot of hT1R2 (c) and hT1R3 (d)

2. Homology Modeling of Bitter Taste Receptor hT2R4

Due to the absence of highly relevant templates in the NCBI database for hT2R4 (with sequence identity below 30%), the I-TASSER online modeling tool (available at https://zhanggroup.org/) was employed for constructing the model. This approach was selected to compensate for the lack of closely related structural templates in traditional databases.

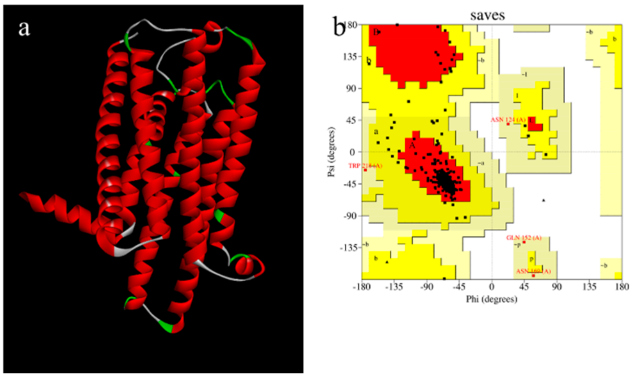

Figure S7. The models and Ramachandran plot of hT2R4

3. Molecular Docking

The docking of the SGs molecules to the receptor was executed using AutoDock Vina 1.5.7 within AMDock(Valdes-Tresanco, Valdes-Tresanco, Valiente, & Moreno, 2020). The calculation of ten most probable binding sites was performed. For hT1R2 and hT1R3, the binding sites are restricted to the VFTD (Venus Flytrap Domain) region. The conformation of SGs with the lowest affinity was then isolated for further analysis (Table S18). The hydrogen bond interactions between SGs and amino acid residues were displayed using PyMOL.

Table S17. The affinity of four steviol glycosides with hT1R2, hT1R3 and hT2R4

	Affinity (kcal/mol)		
	Sweet taste receptors		Bitter taste receptors
	hT1R2	hT1R3	hT2R4
RA	-10.5	-10.2	-9.6
RE	-7.1	-7.9	-6.6

RD	-8.4	-8.8	-6.3
RA1G	-8.1	-7.6	-5.5

Table S18. The key amino acids in sweet receptors interacted with SGs.

hT1R2				hT1R3			
RA	RE	RD	RA1G	RA	RE	RD	RA1G
GLN-125	VAL-330	GLN-125	GLN-125	ALA-46	GLU-48	ARG-137	GLY-33
VAL-330	PHE-338	GLN-361	VAL-330	GLU-47	ARG-52	GLY-168	ASP-34
ASP-364	GLN-361	ASN-365	ARG-339	GLU-48	ARG-233	LEU-408	SER-266
ASN-365	CYS-632	LEU-631	GLN-361	GLY-50	ASN-386	PHE-435	ARG-550
ASN-368	GLN-634	CYS-632	ASN-365	ARG-52		VAL-437	SER-551
CYS-632				ARG-54		TRP-461	ARG-553
				ARG-233		SER-629	
				GLU-240			
				ARG-247			

Table S19. The key amino acids in bitter receptors interacted with SGs.

hT2R4			
RA	RE	RD	RA1G
ALA-9	ASN-165	LEU-4	LEU-2
SER-13		SER-8	ASN-164
THR-166			THR-166
ASN-169			ASN-169
LEU-271			

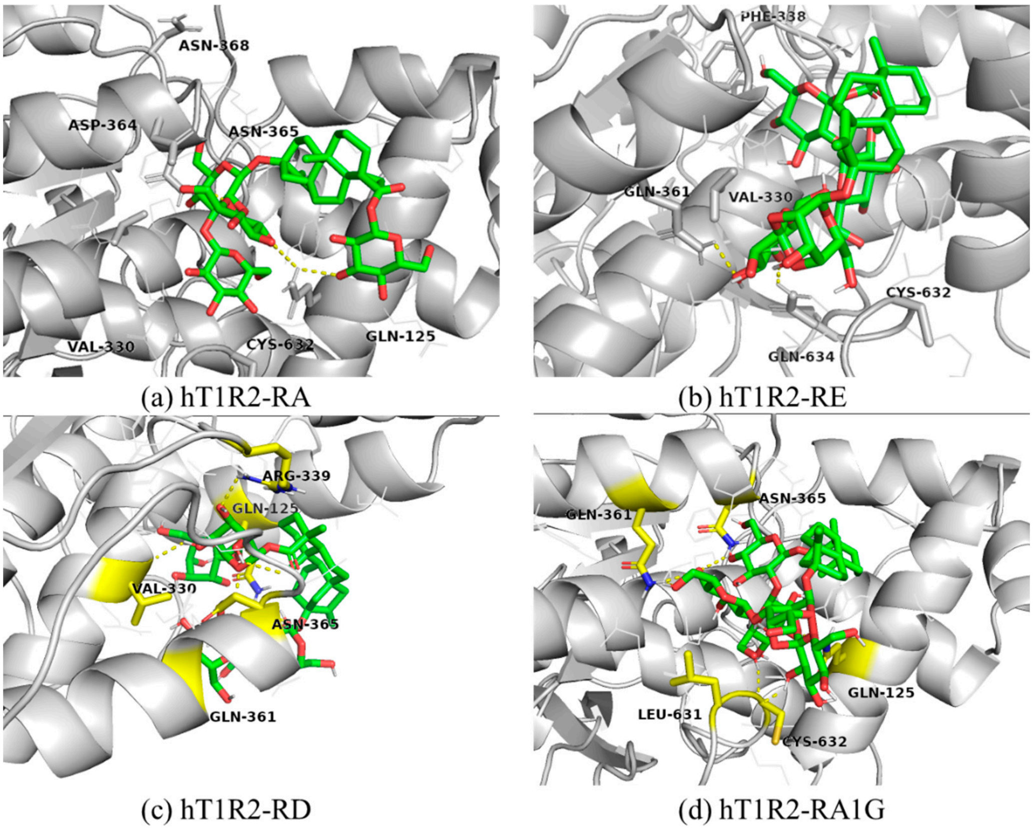

Figure S8. The interaction patterns of four steviol glycosides with hT1R2. Yellow dash: Hydrogen bonds.

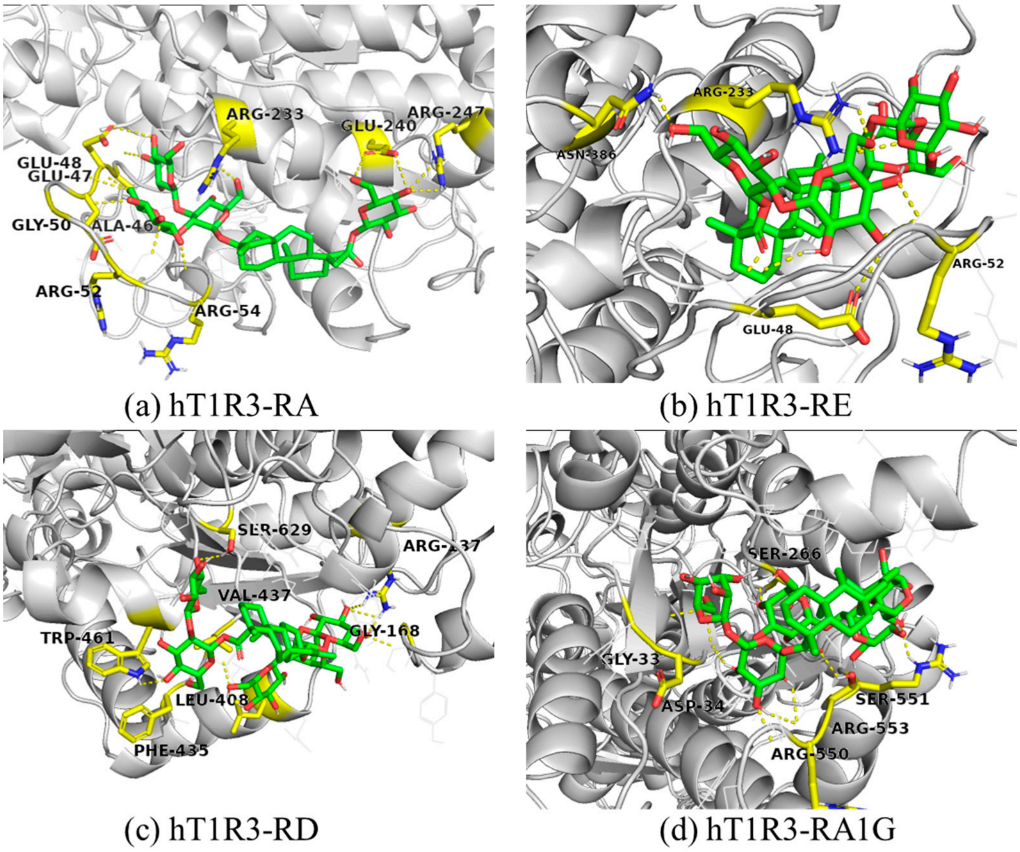

Figure S9. The interaction patterns of four steviol glycosides with hT1R3. Yellow dash: Hydrogen bonds.

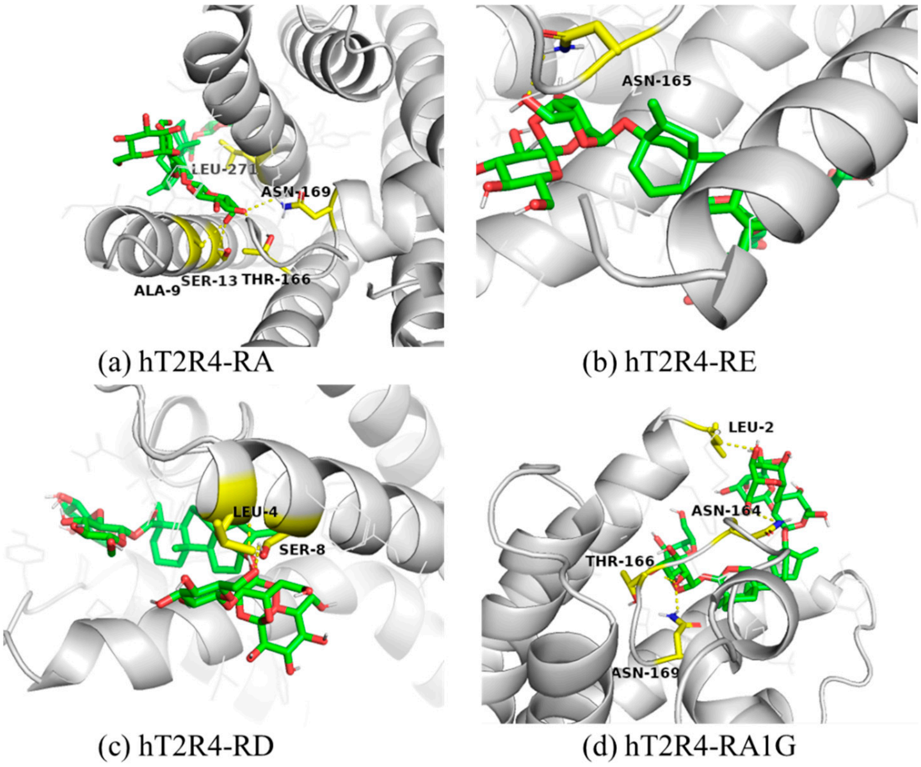

Figure S10. The interaction patterns of four steviol glycosides with hT2R4. Yellow dash: Hydrogen bonds.

References

Kim, S.-K., Chen, Y., Abrol, R., Goddard, W. A., III, & Guthrie, B. (2017). Activation mechanism of the G protein-coupled sweet receptor heterodimer with sweeteners and allosteric agonists. *Proceedings of the National Academy of Sciences of the United States of America, 114*(10), 2568-2573. https://doi.org/10.1073/pnas.1700001114.

Valdes-Tresanco, M. S., Valdes-Tresanco, M. E., Valiente, P. A., & Moreno, E. (2020). AMDock: a versatile graphical tool for assisting molecular docking with Autodock Vina and Autodock4. *Biology Direct, 15*(1). https://doi.org/10.1186/s13062-020-00267-2.

Zhou, Z., Li, W., Wang, H., & Xia, Y. (2024). A Computational Approach to Understanding and Predicting the Edulcorant Profile of Glucosyl Steviol Glycosides. *Foods, 13*(12), 1798. https://doi.org/10.3390/foods13121798.
